# Dyadic nonverbal synchrony during pre and post music therapy interventions and its relationship to self-reported therapy readiness

**DOI:** 10.3389/fnhum.2022.912729

**Published:** 2022-09-06

**Authors:** Sun Sun Yap, Fabian T. Ramseyer, Jörg Fachner, Clemens Maidhof, Wolfgang Tschacher, Gerhard Tucek

**Affiliations:** ^1^Department of Health Sciences, Institute of Health Sciences and Midwifery, IMC University of Applied Sciences Krems, Krems an der Donau, Austria; ^2^Josef Ressel Centre for Horizons of Personalised Music Therapy, IMC University of Applied Sciences Krems, Krems an der Donau, Austria; ^3^Cambridge Institute for Music Therapy Research, Anglia Ruskin University, Cambridge, United Kingdom; ^4^Department of Clinical Psychology and Psychotherapy, Institute of Psychology, University of Bern, Bern, Switzerland; ^5^Department of Experimental Psychology, University Hospital of Psychiatry and Psychotherapy, Bern, Switzerland

**Keywords:** neurological rehabilitation, therapy readiness, therapeutic relationship, motion energy analysis, nonverbal synchrony, music therapy

## Abstract

Nonverbal interpersonal synchronization has been established as an important factor in therapeutic relationships, and the differentiation of who leads the interaction appears to provide further important information. We investigated nonverbal synchrony – quantified as the coordination of body movement between patient and therapist. This was observed in music therapy dyads, while engaged in verbal interaction before and after a music intervention in the session. We further examined associations with patients’ self-reported therapy readiness at the beginning of the session. Eleven neurological in-patients participated in this study. Our results showed an increase in both nonverbal synchrony and patient leading after the music intervention. A significant negative correlation was found between self-reported therapy readiness and nonverbal synchrony after the music intervention. These findings point to the empathic ability of the music therapist to sense patients’ therapy readiness. Higher patient leading in nonverbal synchrony after the music intervention may thus indicate that the music intervention may have allowed dyadic entrainment to take place, potentially increasing self-regulation and thus empowering patients.

## Introduction

Nonverbal synchrony plays an important role in the therapeutic relationship in psychological counseling and psychotherapy (Ramseyer and Tschacher, 2006, 2011; Altmann et al., 2020; Cohen et al., 2021). Music therapy is not only closely related to psychotherapy, but it also takes place within a therapeutic relationship (Österreichischer Berufsverband der MusiktherapeutInnen, 2022). Indeed, the therapeutic relationship is a crucial part of the therapeutic process, 30% of the client’s improvement is accounted for by the therapeutic relationship (Asay and Lambert, 1999). Therefore, it is of great interest to music therapists to be able to capture nonverbal synchrony during their therapy sessions to deepen their understanding of their therapeutic relationships with patients. For this reason, we investigated the phenomenon of nonverbal synchrony during music therapy sessions between therapeutic dyads. Specifically, by comparing nonverbal synchrony, which we defined as quantified coordination of body movement between patient and therapist, while they engaged in verbal interaction before and after a music intervention, using a novel method in music therapy research such as Motion Energy Analysis. Our present study also sought to establish possible associations between nonverbal synchrony and patients’ self-reported therapy readiness.

## Conceptual background

### Synchrony

The world we live in is fond of synchrony. In nature, we observe synchrony as we watch the wonderfully coordinated dance of swarms of birds returning to their nests at dusk. At night, by the tidal rivers of Southeast Asia, thousands of male fireflies congregate in trees, exhibiting in harmony their mating call; a mesmerizing silent concert of synchronized pulsating lights along the water (Strogatz and Stewart, 1993). Even deep inside our body, cells of our hearts generate an electrical rhythm in unison so as to command our hearts to beat, repeating this synchronized action for billions of times in our life time (Strogatz, 2015). In the 17th century, the Dutch astronomer and great inventor, Christiaan Huygens observed and described the phenomenon of the “Sympathy of two clocks”. Forced to stay in bed due to illness, he observed the synchronization of two pendulum clocks which were hanging on a wooden beam. Even after he had disturbed them, they would eventually come to tick simultaneously again. Both clocks were apparently coupled to each other through a free medium, which was the flexible wooden beam, and therefore showed mutual synchronization (Pikovsky et al., 2001).

The word “synchrony” stems from the Ancient Greek words “chronos” (meaning time) and “syn” (meaning common). Taken together, synchrony translates to “occurring at the same time” or “sharing the common time”; an object adjusting its rhythm in conformity with the rhythm of other objects (Pikovsky et al., 2001). In music, “synchrony” could mean a period of phase-locking to an external referent, such as a metronome (Lindenberger et al., 2009). However, when two musicians play together, both interact and adjust toward each other to fit into the created or agreed tempo, creating a common phase which they both “lock in” to, synchronizing electrical brain activity as evidenced in dual EEG studies (Lindenberger et al., 2009). The process of getting “in synch” is described as “entrainment”, it is a process in which two different rhythmic systems adjust to each other although the entraining rhythms do not match precisely (Clayton et al., 2004). Instead, their relationship with each other presents certain regularity (Bluedorn, 2002). The understanding and application of the terms “synchrony” and “entrainment” are not universal at all. Many terminologies have been applied to describe the interdependence behaviors of dyadic partners, such as the chameleon effect, mimicry, behavior matching, attunement, social resonance, synchrony, etc. (Delaherche et al., 2012; Chartrand and Lakin, 2013). The usage in therapy, anthropology, communication studies, etc., varies, depending on the context and the application in the different disciplines (Gill, 2012).

### Nonverbal synchrony

Similar to the two clocks hanging on a single beam, when we observe another person or interact with them, we tend to adopt the physiology and behaviors matching his or her affective state (Stephens et al., 2010; Ebisch et al., 2012). The automatic mirroring of behaviors or the unconscious alignment of physiology during interactions is also called interpersonal synchronization (Feldman, 2007). Referring to Condon and Sander (1974) frame by frame analysis of mother–infant interaction, Aldridge discussed how talking and listening, co-creation and turn-taking find an analog nonverbal structure in music therapy improvisation (Aldridge, 1989).

This coordinative interaction helps us in our daily dealings and is integrated into multiple facets of our social lives (Kendon et al., 1975; Hu et al., 2022). It is a fundamental capacity, essential for us to be social (Cross, 2005). It existed as a main form of communication before language came into being and functions as a “social glue” (Lakin et al., 2003), bringing harmony to relationships and creating bonds. Infants engage in proto-conversation by using their congenital ability to synchronize with the impulses in the action of the parent’s voice and gesture (Trevarthen, 2008). This skill is trained and honed while engaging in “motherese”, an ability developing out of infant-directed speech synchronizing movement and nonverbal elements of vocalizing during “baby-talk” that would give the child a stronger bonding with the mother/carers and a higher likelihood of survival in adulthood. It seems that evolution has permanently engraved these nonverbal abilities to entrain and synchronize with others in us, making them instinctive and unconsciously coordinated when we interact with one another (Lakin et al., 2003; Gueguen et al., 2009; Gill, 2012).

Nonverbal interpersonal synchronization is regarded as an important factor in psychological counseling and psychotherapy related to building rapport and empathy (Kodama et al., 2018; Wiltshire et al., 2020). It plays a quintessential role in the development and maintenance of a positive therapeutic relationship (Ramseyer and Tschacher, 2006; Altmann et al., 2020; Cohen et al., 2021). Patients rate sessions with increased nonverbal synchrony as having higher relationship quality. Nonverbal synchrony is also higher with patients experiencing high self-efficacy and higher symptom reduction at the end of treatment (Ramseyer and Tschacher, 2011).

Music therapy research on nonverbal synchrony between dyads has focused predominantly on the population of autistic spectrum and neonatology. Indeed, nonverbal synchrony is an important indicator of the social and communication skills of children with Autism Spectrum Disorder and severe multiple disabilities (Neugebauer and Aldridge, 1998; Venuti et al., 2017; Johanne and Holck, 2020). It also plays a central role in music therapy with prematurely born infants to reduce stress (Haslbeck, 2014).

In a neurorehabilitation setting, Street et al. (2019) found indications that the auditory-motor pathway can be potentially fortified when the external auditory cues are internalized and patients are able to synchronize their movements independently. Other studies using Rhythmic Auditory Stimulation, which is also based on auditory-motor synchronization, have demonstrated its high success in gait training for various neuropathological gait disorders (Thaut and Abiru, 2010; Braun Janzen et al., 2022). Studies have also supported the efficacy of music therapy on mood, depressive syndromes, and the quality of life of neurological patients (Raglio, 2015). However, there has been so far no research in music therapy exploring nonverbal synchrony in the neurorehabilitation setting that uses state-of-the-art methods that are already established in current psychotherapy research.

Just as it is vital in psychotherapy, therapeutic relationship and alliance are also important factors in music therapy treatment (Silverman, 2019). Indeed, therapeutic relationships between music therapy dyads was found to be predictive of generalized clinical changes of symptom severity in children on the autism spectrum (Mössler et al., 2019). Therefore, this study applied methods already adopted in psychotherapy research to examine the nonverbal synchrony between patient and music therapist during verbal communication before and after the music intervention in a neurorehabilitation setting. The study expects to acquire a new perspective to examine therapeutic alliance and relationship in music therapy research to increase knowledge of effective factors in music therapy processes.

The effects of who is leading (driver) and who is following (driven) during synchrony are relevant to the association between nonverbal synchrony and therapeutic success (Altmann et al., 2020). Patient leading during nonverbal synchrony has been associated with patient’s self-efficacy, whereas therapist leading has been associated with their therapeutic alliance (Ramseyer and Tschacher, 2011). Schoenherr et al. (2019a) found that it is crucial in the early phases of therapy for patients to be leading and therapists to be following, as this would indicate that the patient is speaking. This, in turn, was associated with a lower dropout rate. Therapist leading, on the other hand, influences the clinical outcome. Attention should also be given to what was happening in the session during the shifting of leadership (Schoenherr et al., 2019a). As music therapy is based on a process of mutual relation, the possible implications of leading and following are also of interest, although leading and following in a music therapy context can have reverse signs. The therapist may be leading the music therapy process while following the patient’s play, and encouraging them to lead the musical activity (Brown and Pavlicevic, 1996). Based on their study with children with Autism Spectrum Disorder, Mössler et al. (2019) suggested that music therapists should be able to attune musically and emotionally to their client’s way of communicating and relating, so that clients can develop meaningfully by giving them the opportunities to respond and relate.

### Therapy readiness

Readiness can be defined as “patient’s positive attitude and preparedness to enter into a therapeutic relationship for the purpose of resolving problems” (Ogrodniczuk et al., 2009, p. 427), and having therapy readiness indicates being motivated for therapy (Rapp et al., 2007). Constructs such as the five stages of change, taken from the transtheoretical model of change (Prochaska and DiClemente, 1983), or the readiness for therapy questionnaire by Ghomi et al. (2021), are used in mental health care to predict how likely a person is going to complete therapy or achieve their goals. Therapy readiness is closely related to common factors of psychotherapy such as “readiness to change” and “patient engagement”, which are considered predictors of therapeutic success (Tschacher et al., 2014a).

Timing is an important factor for therapy readiness, and there is only one study that investigated when in the cancer treatment process should music therapy begin (von Allmen et al., 2004). However, timing also applies to the time of day and seasons. The effects of circadian patterns and seasonal changes are well known, for example, for people with depression (Germain and Kupfer, 2008; Wirz-Justice, 2008). Their mood is highest in summer and lowest in autumn and winter. Patients feel worst in the morning and get better as the day progresses. It is important to bear this in mind when scheduling music therapy sessions because daytime and our mood influences how we perceive music and also how we perceive ourselves (Brabant and Toiviainen, 2014). It affects our perception of musically-expressed emotions and also the recalling of memories from our lives, which has direct effects on our sense of self and our outlook on life (Fachner et al., 2017). Thus, the efficacy of music therapy could be increased if we can schedule music therapy sessions at the most suitable time. This information can also help music therapists to better interpret the events during a session, and a better understanding of chronopharmacology will also enhance the effect of medication and music therapy (Fachner et al., 2017). The chronobiological approach of considering temporal processes in biology is a holistic one (Balzer, 2009).

The first goal of this study was to analyse the change in nonverbal synchrony between patient and therapist quantified as the coordination of their body movement. We expected to find an increase in nonverbal synchrony after a music therapy intervention compared to synchrony prior to the intervention. Second, we explored the possible correlation between nonverbal synchrony and patients’ self-reported therapy readiness as well as the leading characteristic of synchrony, to deepen our understanding of the meaning of nonverbal synchrony. We expected a positive correlation between pretalk nonverbal synchrony and therapy readiness.

## Materials and methods

### Sample

This nested study was embedded in a larger research project “Assessment of favorable therapy times for patients in neuro-rehabilitation” (Dür et al., 2022), focusing on developing evaluation methods to assess therapy readiness which involved *N* = 60 participants who were in-patients of a neurological rehabilitation facility in lower Austria. Music therapy was not in the standard treatment but it was offered to all participants in the larger project. The main study included an intensive data collection period of 4 days each. All participants in the nested study were recruited according to the inclusion criteria of the larger project: they had a principal neurological diagnosis, were able to verbalize, were able to give their written informed consent, and were between the ages of 18–99. Exclusion criteria were: pre-existing atrial fibrillation or atrial flutter or new onset of atrial fibrillation or flutter during the observation period, implanted pacemaker or defibrillator, contraindications for adhesive electrodes (e.g., allergy, severe psoriasis, etc.), limiting neuropsychological impairments, due to which keeping an activity log and scale-based self-assessment are not possible or the occurrence of a medical (life-threatening) crisis during the study.

Due to ethical and organizational constraints, we obtained a convenience sample of 11 dyads composed of 11 participants (seven females, four males) and one music therapist (female). The participants were between the ages of 39 and 60 (mean age 51 years, *SD* = 6.48), and the music therapist was 43 years old. None of the participants had physical impairments, such as hemiplegia, that would have impeded their movements. See Table 1 for participants details.

**TABLE 1:** Durations of pretalk, music intervention, posttalk, session, type of music intervention and diagnosis of participants.

ID	Pretalk duration hh:mm:ss	Intervention duration hh:mm:ss	Posttalk duration hh:mm:ss	Session duration minutes	Type of music interventions	Diagnosis ICD-10
P018	00:03:03	00:29:45	00:13:28	52.92	Active music making (drums) and receptive music (patient shared music).	M54.5
P019	00:08:51	00:29:08	00:08:24	48.68	Guided relaxation (sound bowls), song writing and performance.	I63.5
P025	00:05:38	00:26:03	00:04:00	40.13	Breath and voice work. Singing and active music playing (patient on drums and MT on ukulele).	M51.1
P028	00:07:46	00:23:59	00:06:46	54.67	Deep listening exercise (sound bowls) and improvisation on the theme “discovery” on harps.	M54.4
P031	00:04:45	00:35:01	00:02:58	47.52	Improvisation on theme “holidays” (patient on chimes and MT on harp) and active music making (drums).	M51.1
P032	00:24:40	00:11:29	00:05:50	53.97	Vocal improvisation (MT on voice and harp).	M51.1
P035	00:04:16	00:20:09	00:07:45	44.93	Improvisation on theme “under the stars” (harps) and free improvisation (harps and voices).	I63.9
P037	00:12:59	00:15:07	00:13:39	49.27	Improvisation “hands seperated and together” (patient on chimes an MT on harp).	M51.1
P047	00:04:44	00:34:32	00:06:28	52.15	Deep listening (sound bowls), voice work and improvisation (harps).	M51.1
P048	00:17:48	00:27:52	00:13:00	62.48	Improvisation on theme “taking time” (patient on sansula and MT on harp) and improvisation (patient with spoken words and MT on sansula).	M51.1
P057	00:12:46	00:28:14	00:09:54	55.32	Receptive music (patient’s choice) and singing (songs from patient’s native country).	M50.0
Mean	00:09:45	00:25:34	00:08:23	51.09		
Max	00:24:40	00:35:01	00:13:39	62.48		
Min	00:03:03	00:11:29	00:02:58	40.13		
SD	00:06:44	00:07:26	00:03:44	5.94		

### Music therapy settings

**FIGURE 1:**
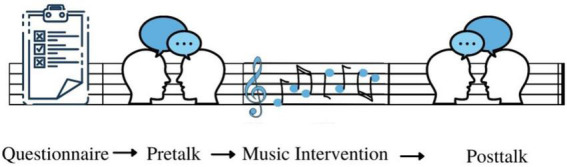
Music therapy session schematic.

The music therapy sessions were conducted in a therapy room. The patient and therapist sat facing each other at about 110° and after they were seated, patient proceeded to fill out a self-reported therapy readiness questionnaire. The music therapy session would start with a conversation (pretalk) to check in on the patient’s condition and to decide on what they would do for the session. This was followed by the music intervention and a subsequent conversation (posttalk) to reflect on the process. As a pragmatic study, it was important to keep the clinical setting as authentic as possible. Thus, the type of music intervention was non-standardized, depending solely on the needs of the patients. It could be receptive music listening, improvisation, active music-making, singing, moving or dancing to music, etc. Likewise, the duration of the music interventions and the unstructured pretalk and posttalk segments during the session were also unrestricted to maintain the organic flow of the therapy sessions. Figure 1 shows the schematic representation of the music therapy sessions. Although music therapy was not a standard treatment, all 11 participants received it during their stay at the hospital. Some continued with music therapy even after the data collection period, totaling between two and five music therapy sessions each. We examined one single session that was between the first and the third music therapy session. The music therapist was trained at IMC University of Applied Sciences Krems (Bachelor and Master) and had worked four years full time in neurology at the time of data collection. Shared moments of musical encounter and the principles of harmonizing psychological and physical states (regulation) are at the heart of the Krems approach to music therapy. In recent years, the anthropological approach in Krems focused on personalization of therapy, leading to the development of new models to facilitate authentic clinical research and abandoning the demand for standardization of therapeutic procedures (Tucek et al., 2021) (see Table 1 for the type of music interventions and diagnoses of participants).

### Video material

The videos of the music therapy sessions were recorded on an iPhone 8 without auto focus at 30 frames per second. Light conditions were held constant, and as a technical demand of the Motion Energy Analysis, the chairs where the dyads were sitting were in fixed positions during pretalk, posttalk, and the music intervention. The camera position was fixed across all sessions. The videos were time-stamped after the recording in Adobe Premiere. The mean duration of the music therapy sessions was 51.09 min (*SD* = 5.94 min, range 40.13–62.48 min). Since the shortest available video was 2 min 58 s (see Table 1 for individual durations), we used 2 min of the verbal interaction immediately before and after the music intervention for all dyads. In nonverbal research in the psychotherapy setting, synchrony tends to decrease over time (Ramseyer and Tschacher, 2011), therefore, we can reject the possibility of any bias in this choice. Furthermore, we explored a dataset with a similar nature, namely data from one single therapist across multiple patients: The author of MEA (Ramseyer, 2020) provides data of himself interacting with *N* = 103 patients during psychotherapy intake-interviews^1^, and from this dataset, we calculated nonverbal synchrony with identical parameters used in the present study. We chose minutes 5–7 and 45–47, in order to gauge a potential temporal effect on nonverbal synchrony. Both 2-min segments had synchronies that did not differ from chance. Pseudosynchrony was equally high in these short time-segments. The comparison of initial minutes (5–7) versus final minutes (45–47) resulted in a significant decrease across time [*T* (102) = 2.057; *p* = 0.04].

### Motion energy analysis

**FIGURE 2:**
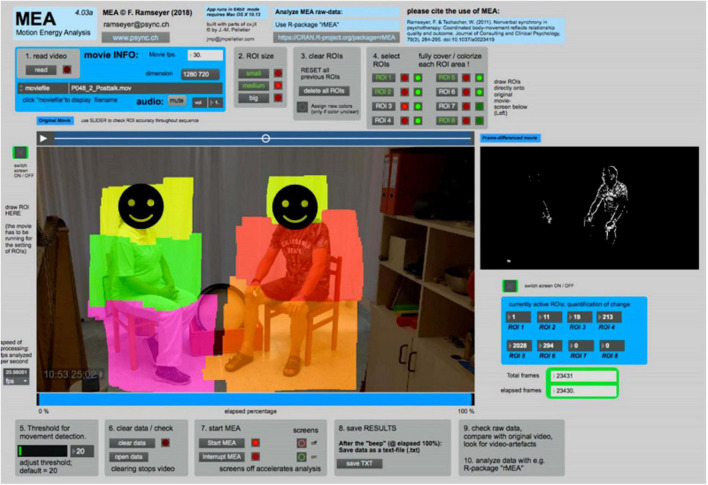
Screenshot of the motion energy analysis (MEA, Version 4.03).

The Motion Energy Analysis (MEA; Ramseyer, 2020) is an automated application that quantifies movements from a video source. By comparing the changes in the pixel of each subsequent frame to its predecessor in predefined regions of interest (ROI), MEA is able to objectively generate time-series of pixel change within ROIs (for details see Ramseyer and Tschacher, 2011; Ramseyer, 2020). Six ROIs were firstly selected in MEA. ROI 1,2, 3, and ROI 4,5,6 were the predefined movement spaces for the head, upper body, and lower body of the music therapist and the patient, correspondingly (Figure 2). Since we are not discriminating movements from different areas of the body in our analysis, we have combined the 3 ROIs for each individual at the next step of analysis based on similar studies using MEA which employed full-body ROIs (Ramseyer and Tschacher, 2011; Erdös and Ramseyer, 2021).

### Calculations of nonverbal synchrony

**FIGURE 3:**
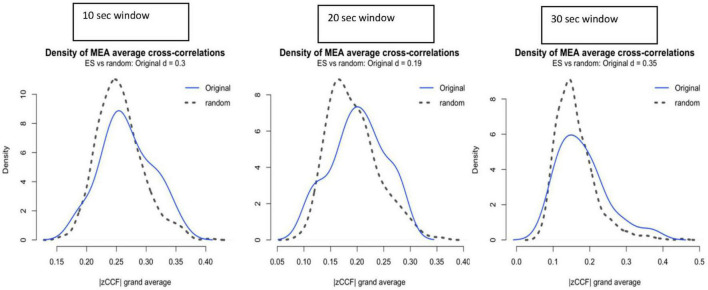
Effect size (ES) of synchrony above pseudosynchrony for window sizes of 10, 20, and 30 s. |zCCF|, Fisher’s Z absolute mean cross-correlation.

To calculate the nonverbal synchrony, the raw data generated by MEA (Ramseyer, 2020) was analyzed with the R package rMEA (R Studio Team, 2020; Kleinbub and Ramseyer, 2021). Using rMEA, the six ROIs were combined into two individual sets, creating two time series, one for the therapist and the other for the patient. Cross-correlation is the most commonly used method for the quantification of nonverbal synchrony in behavioral time series (Boker et al., 2002). The cross-correlations are windowed, i.e., calculated in separate segments, as our data contains human interaction, which is non-stationary. Although the optimal segment size is yet to be determined (Schoenherr et al., 2019b), previous research has used 30 s (Tschacher et al., 2014b) and 60 s (Ramseyer and Tschacher, 2014). Conversely, in another approach of “peak picking”, a segment size of 4 s (Boker et al., 2002) was applied. It has also been suggested that the window width should be at least 50–70 values and about 4–5 times larger than the maximum time lag used (Cappella, 1996). Furthermore, since there is no one-size-fits-all solution, the length of the window is dependent on the processes occurring at different time scales (Moulder et al., 2018). In this study, the length of the selected data is 2 min long. Since we are interested in within-session phenomena, a segment size of 10 s was selected. This segment size is also the best mid value option after having examined the effect size of other segment sizes. Figure 3 shows the comparison of our calculated synchrony to that of the pseudosynchrony. On the X-axis we show the Fisher’s Z absolute mean cross correlation and the Y-axis provides the graphical representation of the distribution of these correlations.

Time series of two individuals are cross correlated at different time lags, thus allowing for possible delayed or lagged associations of movement activity. Similar to the segment size, the time lag is also largely at the discretion of the researcher (Schoenherr et al., 2019b). Previous studies have used ±5 s (Ramseyer and Tschacher, 2014; Tschacher et al., 2014b; Cohen et al., 2021; Erdös and Ramseyer, 2021). Using a lag of ±2 s was considered to be a conservative and suitable measure as real synchrony intercepted random synchrony at close to −0.5 and +2. Therefore, by choosing the value of ±2 s (Figure 3), more power was given to the pseudosynchrony derived from the surrogate test to be equally strong as the real signal, which increases the strength of the test. The windowed cross-correlation in our study was done without overlapping.

### Pseudosynchrony

To ascertain that the synchrony we have observed was not merely a random coincidence, we compared the results to values of pseudosynchrony. Therefore, if actual synchrony is genuine, it should be higher than pseudosynchrony, with a greater effect size (Ramseyer and Tschacher, 2010). The pseudosynchrony was generated by bootstrapping, i.e., recombining one dyad’s movement time series with another random movement time series from the pool of all observations, thus creating surrogate datasets. As suggested by Kleinbub and Ramseyer (2021), we used between subject shuffling, which was also better suited for the rather short duration of the analyzed segments in our sample. Within subject shuffling would have provided insufficient recombinations, if the segment size was kept identical in both real and pseudo interactions (Ramseyer and Tschacher, 2010; Moulder et al., 2018). Three pools of data were used for producing the surrogate datasets. For better results, each pool’s sampling was done without replacement, since the sample size is small. Pool 1 consisted of all possible pretalk and posttalk data combinations for all members of any dyad (*N* = 924, i.e., 22 × 21 × 2), pool 2 consisted of all pretalk data (*N* = 220, i.e., 11 × 10 × 2), and pool 3 consisted of all posttalk data (*N* = 220, i.e., 11 × 10 × 2). The actual pretalk synchrony was compared to pseudosynchrony created by pools 1 and 2. The actual posttalk synchrony was compared to pseudosynchrony created by pools 1 and 3. Thus, the pseudosynchronies were produced by between-shuffling of interactions (Kleinbub and Ramseyer, 2021). Apart from calculating the nonverbal synchrony and comparing these to the pseudosynchrony, the roles of leader and follower were directly accessible through the differentiation into positive and negative lags. This distinction has been used in previous research (Ramseyer and Tschacher, 2011), and it is statistically implemented in the rMEA package (Kleinbub and Ramseyer, 2021). We thus ascertained the roles during the pretalk and posttalk using statistical procedures available in rMEA.

### Correlation between nonverbal synchrony to visual analog scale of patients’ self-reported therapy readiness

Before the commencement of the therapy session, patients were asked to complete a questionnaire regarding their therapy readiness. Their responses were unknown to the music therapist. This questionnaire consisted of 11 items, including a traditional visual analogue scale (VAS) (Dür et al., 2019). The VAS is a type of psychometric scale commonly used to quantify psychological phenomena such as satisfaction, well-being, mood, etc. VAS has been shown to have test-retest reliabilities of at least 80% across different constructs and contexts. In pain or quality of life studies, VAS is also comparable to the established gold standard of measurements (Nguyen and Fabrigar, 2018). The German instruction on the VAS can be translated as “Please use the following visual analog scale to indicate your estimation. Please mark the position on the bar with a vertical line that corresponds to your current therapy readiness”. The left side of the VAS indicated an absolute readiness for therapy, and the right side indicate the contrary. Patients indicated their subjective assessments of their readiness for therapy by marking it on this 10 cm continuum. Items 1–10 of the questionnaire had very low variance, resulting in a ceiling effect. Therefore, only the VAS was used to make a statistical correlation with the nonverbal synchrony, using the application Jamovi 1.8.1.0 (The Jamovi Project, 2021).

## Results

### Nonverbal synchrony versus pseudosynchrony

**FIGURE 4:**
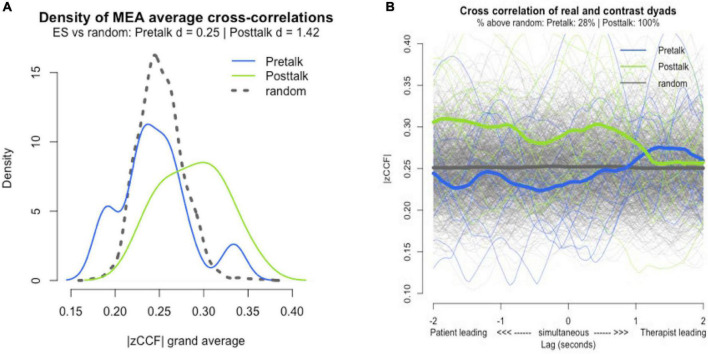
Synchrony vs pseudosynchrony. **(A)** Effect size of pretalk, posttalk, and pseudosynchrony. |zCCF|, Fisher’s Z absolute mean cross-correlation. **(B)** Lag-plot of pretalk and posttalk synchrony. Gray line = pseudosynchrony (random).

We compared the nonverbal synchrony of the dyads during the pretalk and posttalk to pseudosynchronies. This revealed statistically significant differences between posttalk synchrony and pseudosynchrony derived from pool 1 (entire pretalk and posttalk data; *N* = 924, *p* = 0.00976; *d* = 1.42) as well as from pool 3 (posttalk data only; *N* = 220, *p* = 0.02046; *d* = 1.12). Pretalk synchrony did not demonstrate a statistically significant value over the pseudosynchrony (Figure 4).

### Nonverbal synchrony pretalk versus nonverbal synchrony posttalk

**FIGURE 5:**
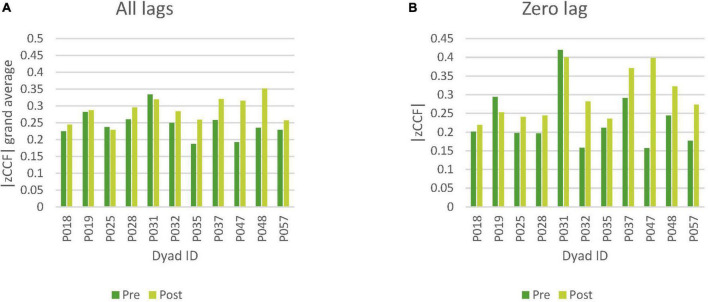
Comparison of pretalk and posttalk nonverbal synchrony. **(A)** Comparison based on all lags values (grand average). **(B)** Comparison based on lag zero values (only lag zero cross-correlations). —zCCF— Fisher’s Z absolute mean cross-correlation.

Comparing nonverbal synchrony in pretalk to nonverbal synchrony in posttalk, we found that nine out of eleven dyads showed an increase in nonverbal synchrony after music intervention for both all lags and lag zero (Figure 5).

Non-parametric test Wilcoxon Signed-Ranks Test indicated that nonverbal synchrony (all lags) was significantly higher in the posttalk (*Mdn* = 0.29) than the pretalk (*Mdn* = 0.24), *W* = 5, *p* = 0.010, *r* = −0.85. Significant increase was also found in the nonverbal synchrony (lag zero) for posttalk (*Mdn* = 0.27) than pretalk (*Mdn* = 0.20) *W* = 6, *p* = 0.014, *r* = −0.82 (Tables 2, 3). For parametric test, please refer to Table 4.

**TABLE 2:** Descriptive.

	Pretalk (all lags)	Posttalk (all lags)	Pretalk (lag zero)	Posttalk (lag zero)	Pretalk Therapist Lead	Pretalk Patient Lead	Pretalk Tlead -Plead	Posttalk Therapist Lead	Posttalk Patient Lead	Posttalk Tlead -Plead	VAS
*N*	11	11	11	11	11	11	11	11	11	11	10
Missing	0	0	0	0	0	0	0	0	0	0	1
Mean	0.245	0.288	0.233	0.295	0.258	0.233	0.0244	0.278	0.297	−0.02	94.2
Median	0.237	0.287	0.202	0.274	0.251	0.229	0.0172	0.291	0.291	−0.03	95.8
Standard deviation	0.041	0.0376	0.0768	0.0673	0.0317	0.0534	0.0322	0.0435	0.0518	0.0592	6.23
Minimum	0.187	0.229	0.157	0.219	0.227	0.147	−0.0188	0.166	0.21	−0.125	82
Maximum	0.334	0.352	0.419	0.4	0.324	0.343	0.0795	0.326	0.379	0.0985	100
Shapiro-Wilk W	0.942	0.97	0.836	0.863	0.882	0.939	0.944	0.817	0.973	0.979	0.871
Shapiro-Wilk p	0.544	0.884	0.028	0.063	0.111	0.507	0.57	0.016	0.917	0.959	0.103

**TABLE 3:** Results of paired samples Wilcoxon test.

		Statistic Wilcoxon W	p-value	Mean Difference	SE Difference	95% Confidence			Effect size
						Lower	Upper		
Pretalk (all lags)	Posttalk (all lags)	5	0.01	−0.037	0.014	−0.07350	−0.00780	Rank biserial correlation	−0.848
Pretalk (lag zero)	Posttalk (lag zero)	6	0.014	−0.0545	0.0229	−0.11090	−0.01425	Rank biserial correlation	−0.818
Pretalk therapist leading	Posttalk therapist leading	17	0.175	−0.0231	0.0152	−0.05900	0.01690	Rank biserial correlation	−0.485
Pretalk patient leading	Posttalk patient leading	0	<0.001	−0.0575	0.017	−0.11170	−0.02925	Rank biserial correlation	−0.1
Pretalk Tlead-Plead	Posttalk Tlead-Plead	59	0.019	0.0439	0.0158	0.00810	0.07945	Rank biserial correlation	0.788

**TABLE 4:** Results of parametric paired sample *T*-tests.

		Student’s *t*	df	*p*
Pretalk (all lags)	Posttalk (all lags)	−3.03	10	0.013
Pretalk (lag zero)	Posttalk (lag zero)	−2.69	10	0.023
Pretalk therapist leading	Posttalk therapist leading	−1.33	10	0.212
Pretalk patient leading	Posttalk patient leading	−3.78	10	0.004
Pretalk Tlead-Plead	Posttalk Tlead-Plead	2.78	10	0.02

### Leading in pretalk and posttalk synchrony

**FIGURE 6:**
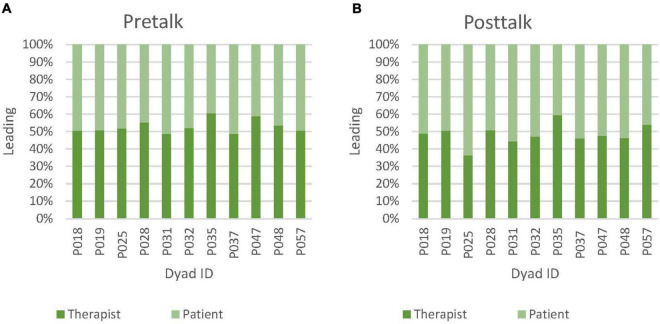
**(A)** Relative amounts (percentages) of therapist leading vs patient leading in pretalk nonverbal synchrony. **(B)** Relative amounts (percentages) of therapist leading vs patient leading in posttalk nonverbal synchrony.

Further inspections revealed that during pretalk, therapist leading was higher than patient leading in 9 out of 11 cases (Figure 6). However, in posttalk, patient was leading in 7 out of 11 cases.

A non-parametric test also showed that the increase in posttalk patient leading (*Mdn* = 0.29) from pretalk patient leading (*Mdn* = 0.23) was statistically significant *W* = 0, *p* < 0.001, *r* = −1.00. Pretalk therapist leading minus patient leading (Tlead-Plead) (*Mdn* = 0.02) was also significantly different to posttalk (Tlead-Plead) (*Mdn* = −0.03), *W* = 59, *p* = 0.019, *r* = 0.788 (Tables 2, 3). For parametric test, please refer to Table 4.

### Visual analog scale of patient’s self-reported therapy readiness

Patients’ therapy readiness ranged between 82 and 100% (*n* = 10, *M* = 94.2%, *Mdn* = 95.8%, *SD* = 6.23).

### Association of nonverbal synchrony with patient’s self-reported therapy readiness

Simple linear regression was calculated to predict pretalk and posttalk nonverbal synchrony as well as therapist leading and patient leading during pretalk and posttalk, based on patients’ self-reported therapy readiness. A significant negative correlation was found between patients’ self-reported therapy readiness and posttalk synchrony (all lags) [*F*(1,8) = 5.32, *p* = 0.050, with an *R*^2^ = 0.40] as well as a trending negative correlation between patients’ self-reported therapy readiness and patient leading during posttalk synchrony [*F*(1,8) = 3.83, *p* = 0.086), with an *R*^2^ = 0.324]. Otherwise, no further associations were found (Table 5).

**TABLE 5:** Correlations between nonverbal synchrony and patients’ self-reported therapy readiness.

	*F*	df 1	df 2	*p*	*R* ^2^	*t*
Pretalk (all lags)	5.9	1	8	0.465	0.0686	−0.768
Pretalk (lag zero)	3.32	1	8	0.106	0.293	−1.82
Posttalk (all lags)	5.32	1	8	0.05	0.4	−2.31
Posttalk (lag zero)	1.22	1	8	0.302	0.132	−1.1
Posttalk minus pretalk (all lags)	1.11	1	8	0.323	0.122	−1.05
Posttalk minus pretalk (lag zero)	1.42	1	8	0.268	0.15	1.19
Pretalk therapist leading	0.28	1	8	0.611	0.0399	−0.529
Posttalk therapist leading	1.57	1	8	0.246	0.164	−1.25
Pretalk patient leading	0.701	1	8	0.427	0.0806	−0.837
Posttalk patient leading	3.83	1	8	0.086	0.324	−1.96

## Discussion

In summary, a statistically solid signal of synchrony with a large effect size (*d* = 1.42) only emerged in the posttalk, suggesting that nonverbal synchrony was only relevant during posttalk. Even though we cannot ascertain a level of synchrony beyond the level of coincidence in the pretalk, we have to bear in mind that the randomness of the pseudosynchrony concept itself has weaknesses: it works best in conditions where time series do not show specific, time-locked (repetitive) patterns. For example, if we were to compare the synchrony of two pendulums, pseudosynchrony would be equally high as genuine synchrony, because the different pendulums would still swing at the same frequencies (Ramseyer and Tschacher, 2010). In our study, all the sessions were conducted by the same music therapist, hence the potential for a certain degree of periodicity was embedded in the data, as her idiosyncratic movements would probably be constant across all sessions. Besides, due to the small sample size (*N* = 11), pseudosynchrony was expected to be rather close to the real synchrony, considering that only limited permutations were possible in the bootstrapping procedure due to the limited number of possible recombinations. Nevertheless, since the average pretalk synchrony (all lags) is 0.24 and the average posttalk synchrony (all lags) is 0.29, there is a strong case for the increase in synchrony even though nonverbal synchrony during pretalk was not beyond pseudosynchrony. Although there was no control group in this study, the observed synchrony was compared with pseudosynchrony (*N* = 924, *N* = 220), which indicated that the posttalk synchrony is beyond chance. A similar synchrony study using MEA revealed that dyads showed no increase in synchrony between two unstructured conversations of 6 mins each, with a filler task of watching a 6 mins film (Fujiwara et al., 2019).

In this study, the analysis of nonverbal synchrony within single music therapy sessions revealed a change in the level of nonverbal synchrony after music intervention. This increase in nonverbal synchrony during posttalk could suggest that the music intervention, akin to the flexible wooden beam in Huygen’s observation, has served as a platform where entrainment took place. Entrainment is a delicate process and as shown by Huygen’s clocks, the intensity of coupling should not be so strong that the individuality is lost (Bennett et al., 2002; Clayton et al., 2004). The music intervention seemed to have given enough space for the dyads to communicate with each other, reacting and moving to adapt to entrain to each other’s rhythm as measured with motion energy during conversation. The increase in nonverbal synchrony after the music intervention may have been driven by increasing familiarity between the therapist and the patient, for having shared some time together. However, other studies point in the other direction, indicating that familiarity could also be causing a decrease of nonverbal synchrony (Erdös and Jansen, 2022).

With this study, we have also established a novel method in music therapy research, using Motion Energy Analysis in the pretalk and posttalk to capture and objectively quantify the process of entrainment in movement behaviors of patients and therapists.

Although our study has shown an increase in nonverbal synchrony after music intervention, the question of the meaning of this increase or whether this increase is necessarily beneficial still remains unanswered. It is important to look at nonverbal synchrony with a more differentiated view as recent research has shown that nonverbal synchrony is a multidimensional construct. Behaving synchronously has been strongly associated with positive aspects of social relationships (Chartrand and Lakin, 2013). Nonverbal synchrony has been associated with positive therapeutic relationships (Ramseyer and Tschacher, 2011; Altmann et al., 2020; Cohen et al., 2021), higher efficacy and higher symptom reduction at the end of treatment (Ramseyer and Tschacher, 2011), and low nonverbal synchrony has also predicted premature termination of therapy (Paulick et al., 2018). This is especially so with sessions at the beginning of the therapy process, as it may indicate low therapy alliance or an ill match between the therapeutic dyads (Schoenherr et al., 2019a). It has also been suggested that social interaction dynamics have a greater effect on our self-regulation than our own individual processes (Galbusera et al., 2019). Whilst these studies have shown that nonverbal synchrony is associated with positive benefits, others have found only marginally significant association (Schoenherr et al., 2019a) or even none at all. One interpretation of these conflicting results focuses on the varying methods for the statistical quantification of nonverbal synchrony (Paulick et al., 2018; Lutz et al., 2020; Ramseyer, 2020).

Beyond the method of analysis, higher levels of nonverbal synchrony may not be equatable to better therapy outcomes. A medium level of nonverbal synchrony between therapeutic dyads was associated with successful therapies where patients improved. Surprisingly, therapies were consensually terminated with no improvements for dyads with the highest level of nonverbal synchrony. At the same time, patients dropped out with no improvement for the lowest level of nonverbal synchrony (Paulick et al., 2018). In addition, a downward trend of nonverbal synchrony cannot be interpreted as a deterioration of therapeutic alliance, as one of the first studies employing MEA found a slight decrease in synchrony from the initial third of therapies to the final third (Ramseyer and Tschacher, 2011). An international coaching study with high level of client reported success (Erdös and Ramseyer, 2021) found that nonverbal synchrony showed a linear trend for a temporal decrease across ten sessions of coaching. It found no overall correlation between nonverbal synchrony and working alliance, affect balance, and goal attainment as such, but a temporal network analysis indicated differential associations in subgroups of higher or lower success. At the beginning of the entire group, synchrony was not indicative of a good coaching alliance. It appeared to be acting as a “corrective mechanism” in dyads with less stable working alliances. Thus, higher levels of nonverbal synchrony in these cases may be interpreted as emerging efforts to correct the process, to attain “the same wavelength with each other,” which subsequently became less necessary when the progress was successful. Dyads do not maintain a constant level of synchrony, instead, they move in and out of interpersonal synchrony (Erdös and Ramseyer, 2021). This aspect of “effort” stands in line with another finding from the domain of psychotherapy, where so-called ruptures in the alliance were followed by higher synchrony (Deres-Cohen et al., 2021). A similar phenomenon has been found in physician-patient interactions in the oncology setting: dyads with a white physician and a black patient displayed significantly higher nonverbal synchrony in comparison with dyads comprised of racially-concordant members (Hamel et al., 2022).

Other factors, such as the pathology of the patients, the body responsiveness of the therapist, dyad type, and therapeutic approaches also appears to affect the level of nonverbal synchrony in session (Schoenherr et al., 2019a).

### Finding on leading and following

Although it is not clear how this should be translated in the case of looking at processes occurring during a single session, such as what we are looking at in our study, we can infer that posttalk synchrony is most likely influenced by the music intervention. So, if we move to the discussion about changes in leading/following after the music intervention, we observed that patient leading in nonverbal synchrony increased significantly when comparing posttalk to pretalk. Another significant difference was found when comparing the difference between therapist leading and patient leading (Tlead – Plead) during pretalk (*Mdn* = 0.02) and posttalk (*Mdn* = −0.03).

Schoenherr et al. (2019a) found that in the early phases of therapy patients leading while therapists follow can affect drop-out rates. This would indicate that the patient is speaking, hence also moving. Therapist leading, on the other hand, influences the clinical outcome. The different leading and pacing appear to have influenced the outcomes in various ways (Altmann et al., 2020). Moreover, attention should also be given to what was happening in the session during the shifting of leadership (Schoenherr et al., 2019a). A recent study had found that different types of musical interaction influenced the subsequent dyadic interaction. Turn-taking music making behavior, for example, was manifested in the heightened attunement during the post music mirror game and more switching of leading/pacing (Buchkowski, 2018). This could be an explanation for the switch in leadership in our study. The significant increase in patient leading in nonverbal synchrony after the music intervention may indicate that dyadic entrainment during the music intervention had potentially increased self-regulation and empowered patients, which led to an increase in the patient leading in the nonverbal synchrony during the posttalk.

### Finding on correlations between readiness and nonverbal synchrony

In this study, we were expecting a positive correlation between patients’ self-reported therapy readiness and pretalk nonverbal synchrony. We did not find a positive significant correlation; instead, we found a non-significant negative correlation. Moreover, a significant negative correlation was shown between patients’ self-reported therapy readiness and posttalk nonverbal synchrony (all lags), as well as a non-significant (*p* = 0.086) but trending correlation with patient leading during nonverbal synchrony in posttalk was shown.

These results are only counterintuitive so far as therapy readiness and nonverbal synchrony are conceptualized as favorable aspects: Therapy readiness may be driven by a high level of suffering or pathology, which could lead to a therapist’s high level of mastery interventions, as shown in a psychotherapy context. Furthermore, nonverbal synchrony may be a sign of a high effort/engagement from the therapist (Deres-Cohen et al., 2021; Erdös and Ramseyer, 2021).

Regarding the negative correlation between patients’ self-reported therapy readiness and posttalk nonverbal synchrony: the readiness data was collected at the beginning of the session, and we were not necessarily expecting a positive correlation because once the therapy was over, why should patients still be ready for therapy? The therapy has already passed, and thus decrease in therapy readiness was most likely being reflected in the negative correlation.

Another speculation could be that although the music therapist was unaware of the patients’ self-reported therapy readiness, she was able to perceive the ambiguous attitude. This could influence the therapist to make extra cheerleading-like efforts to engage the patient during the pretalk, which may be reflected by higher therapist leading than patient leading. This cheerleading effort may not have been necessary any more during the posttalk, and that was subsequently reflected in the posttalk synchrony as an increase in patient leading.

We could also consider that patients’ self-reported therapy readiness may have fluctuated during the session, or may be generally less connected with nonverbal synchrony than for example alliance. However, if patients’ self-reported therapy readiness did have an influence on the level of nonverbal synchrony, we would expect to see a significant positive correlation to the pretalk synchrony and to the posttalk synchrony. This was not the case, probably because our sample size was not strong enough to allow for a conclusive relationship between the pretalk synchrony and patients’ self-reported therapy readiness. Hence, at this point, our study does not reveal whether patients’ self-reported therapy readiness was a good predictor of nonverbal synchrony.

### Strengths and limitations

Since this is a convenience sample with a small sample size, the results should not be generalized, and the results should be cautiously interpreted as they are susceptible to bias. The sample size not only impeded any generalization of the findings but also lowered the possibility of more significant correlations. Pseudosynchrony was also closer to actual synchrony due to the small sample size and also because of the aforementioned weakness of having the same music therapist across all dyads.

Another limitation concerning the music therapist is that she may also have an experimenter bias in her dual role as the researcher as well. Nevertheless, the knowledge that nonverbal synchrony is being assessed need not necessarily increase the phenomenon. The assessment of nonverbal synchrony draws on an attribute that is outside conscious control as data presented in a recent study on the use of MEA has suggested. Knowing that synchrony is being assessed did not increase the amount of synchrony displayed, in fact, synchrony decreased as the therapist’s professional experience augmented (Ramseyer, 2020).

Since the patients who participated in the study were highly motivated, we did not have any representation on the low end of the scale (patient was not ready for therapy). This ceiling effect resulted in less variance in our statistics.

Although patients were asked to indicate their level of therapy readiness, they may not have interpreted the meaning of “ready for therapy” in the same way. The VAS is a cognitive measure that may not be able to capture the difference between the bodily state of “being ready” for therapy and the cognitive appraisal of readiness. This could be a reason for the lack of correlation between patients’ self-reported therapy readiness and the nonverbal synchrony and the leading characteristics. Patients themselves may not have known how ready they actually were at the beginning of the session. A patient’s indication might be considered as low in this sample, but that could also be the highest value he or she would have given, compared to other therapies and at other times. There is no objective measurement of this variable. In addition, patients could have been “ready” for music therapy because they were exhausted and were looking forward to a relaxation exercise. They would perhaps have indicated to be less ready if they were going to another type of therapy session.

The MEA application is a simple, user-friendly tool that quantifies the dynamics of movement irrespective of movement quality. A recent study confirmed the similarity with more sophisticated measures such as OpenPose (Fujiwara and Yokomitsu, 2021). Still, MEA does not qualitatively assess the movements. It does not tell us if the movement was a smile or a roll of the eyes but these types of movements can be pivotal to the relationship. Furthermore, MEA does not measure nonverbal synchrony when there are no movements. This stillness could be actually more meaningful than moving. Nevertheless, MEA provides us an accessible objective, and quantifiable way to examine certain aspects of therapeutic interactions which can support and communicate the efficacy of the therapy. It is also an additional behavioral marker quantifying the observation of the therapeutic process.

### Conclusion and future directions

This study presented a novel way to investigate nonverbal synchrony in music therapy in the neuro-rehabilitation setting. We found increased nonverbal synchrony and patient leading synchrony after the music intervention, which could indicate a growing bond between the dyads through the entrainment during music interventions. Further, this study reports a negative correlation between patients’ self-reported therapy readiness and post music intervention nonverbal synchrony and its characteristics. Future studies should strive for a bigger sample size with different pairs of dyads so that it would be possible to make a generalization and remove possible bias. Data should be collected across multiple sessions across longer time to investigate possible temporal changes.

Different standardized assessments for symptom reduction, therapeutic relationship quality, or mood change could also be included to study what an increase in nonverbal synchrony in the music therapy settings with neuro-rehabilitation patients could indicate.

Sessions should also be scheduled at different times of the day for the individuals, to gather data from different states of therapy readiness, and to look deeper into the chronobiological effects.

This study utilized a combined ROI, but ROIs could also be separated between head and body, to examine nonverbal synchrony with more details. Future research could also look deeper into segments of the music interventions that had taken place to establish possible relation to nonverbal synchrony. Therapists’ assessments of patients’ readiness could also be compared to patients’ self-reported therapy readiness to investigate the effects of therapists’ expectations.

## Data availability statement

The raw data supporting the conclusions of this article will be made available by the authors, without undue reservation.

## Ethics statement

The studies involving human participants were reviewed and approved by Ethics Commission of Lower Austria GS1-EK-4/633-2020. The patients/participants provided their written informed consent to participate in this study.

## Author contributions

SSY carried out the therapies, acquired, and prepared the data. SSY and FR performed the synchrony calculations and the initial analysis of the data. JF, CM, GT, and WT supervised the project. SSY drafted the manuscript. All authors conceptualized and designed the study, discussed the results, revised the manuscript critically, and approved the submitted version of the manuscript.

## Footnotes

1
https://osf.io/gkzs3/
